# Exploring Families’ Acceptance of Wearable Activity Trackers: A Mixed-Methods Study

**DOI:** 10.3390/ijerph19063472

**Published:** 2022-03-15

**Authors:** Amy V. Creaser, Jennifer Hall, Silvia Costa, Daniel D. Bingham, Stacy A. Clemes

**Affiliations:** 1 School of Sport, Exercise, and Health Sciences, Loughborough University, Loughborough LE11 3TU, UK; a.creaser@lboro.ac.uk (A.V.C.); s.costa@lboro.ac.uk (S.C.); 2 Bradford Institute for Health Research, Bradford Teaching Hospitals Foundation Trust, Bradford BD9 6RJ, UK; jennifer.hall@bthft.nhs.uk (J.H.); daniel.bingham@bthft.nhs.uk (D.D.B.); 3 National Institute for Health Research (NIHR) Leicester Biomedical Research Centre, University Hospitals of Leicester NHS Trust, University of Leicester, Leicester LE5 4PW, UK

**Keywords:** Fitbit, pillar integration process, technology acceptance model, theoretical domains framework, feasibility

## Abstract

Background: The family environment plays a crucial role in child physical activity (PA). Wearable activity trackers (wearables) show potential for increasing children’s PA; however, few studies have explored families’ acceptance of wearables. This study investigated the acceptability of using wearables in a family setting, aligning experiences with components of the Technology Acceptance Model and Theoretical Domains Framework. Methods: Twenty-four families, with children aged 5–9 years, took part in a 5-week study, where all members were provided with a Fitbit Alta HR for 4 weeks. Acceptability was measured using weekly surveys and pre-post-questionnaires. Nineteen families participated in a focus group. Quantitative and qualitative data were integrated using the Pillar Integration Process technique. Results: Pillars reflected (1) external variables impacting wearable use and PA and (2) wearable use, (3) ease of use, (4) usefulness for increasing PA and other health outcomes, (5) attitudes, and (6) intention to use a wearable, including future intervention suggestions. Conclusions: Families found the Fitbit easy to use and acceptable, but use varied, and perceived impact on PA were mixed, with external variables contributing towards this. This study provides insights into how wearables may be integrated into family-based PA interventions and highlights barriers and facilitators of family wearable use.

## 1. Introduction

Physical activity (PA) levels of parents and their children are correlated [1], and children with siblings tend to have higher levels of PA in comparison to children without siblings [2,3]. The family environment (e.g., parental attitudes, modelling, encouragement, child autonomy) contributes towards child PA levels [4,5] and outdoor play [6]. Thus, the family environment is a crucial setting for the promotion of active behaviours in children, and this has been emphasized further during the COVID-19 pandemic [7,8,9].

### 1.1. Physical Activity Interventions

Family-based interventions have, to date, offered small effects on child PA [10]. A realist synthesis found that behaviour-change techniques (BCTs), such as goal setting and reinforcement, may be effective mechanisms of action for increasing child PA within the family [10], mediating the relationship between family constraints’ (e.g., time) and child PA levels [10]. Wearable activity trackers (wearables) incorporate goal setting and reinforcement as well as other effective BCTs, such as feedback, self-monitoring, and action planning [11], which align with mechanisms of action underlying effective family-based interventions [10].

There has been an increase in studies investigating the acceptability, feasibility, and effectiveness of wearables in younger populations (5 to 19 years), with some finding they can increase step counts and moderate-to-vigorous-intensity PA (MVPA) [12,13]. Despite this, there is little research investigating the use, acceptability, feasibility, and/or effectiveness of wearables within the family [13]. A recent systematic review [13] identified two intervention studies (targeting adolescents) [14,15] and one feasibility study [16] using wearables in a family setting [13]. There was no evidence that family-based wearable interventions were effective at increasing adolescent PA [14,15], but parents reported becoming more aware of their child’s PA levels and having more conversations about health as a result of the wearable [16]. Since this systematic review, a multi-component intervention (Step It Up Family), which incorporates wearables (Garmin Vivofit 3 and Vivofit jr), increased MVPA in children (6 to 10 years) and their mothers and fathers [17]. This offers some insight into the potential effectiveness of wearables for increasing younger children (vs. adolescents) and parent PA. Despite this, several barriers of children/adolescents using wearables have been identified [13], and it is still unclear how wearables can be best utilised within the family environment. Given the cost (financial resources and time) of interventions to develop, implement, and evaluate, the importance of conducting acceptability or feasibility studies to inform large-scale interventions are widely documented [18,19,20]. Exploring the acceptability of proposed intervention tools can anticipate barriers to future intervention implementation and effectiveness [21].

### 1.2. Theoretical Frameworks

The use of theoretical frameworks has been highlighted as a key way to develop interventions, understand mechanisms of action, and enhance public health practices [22,23]. The Technology Acceptance Model (TAM [24]) and Theoretical Domains Framework (TDF [25]) are two frameworks that can be used to understand the acceptance of wearables (TAM), including their barriers and facilitators of impacting PA (TDF), to inform future interventions.

The TAM [24] can be used to explain an individual’s acceptance of technology. The TAM suggests that external variables impact the perceived ease of use and usefulness of technology, which then impacts the behavioural intention to use the technology either directly (usefulness) or indirectly (ease of use and usefulness) via attitudes [24]. It is suggested that these components then influence actual technology use [24]. There is evidence that the TAM can explain wearables use in adults [26,27,28,29]. However, only one identified study has used the TAM to explore wearable acceptance in individuals under the age of 18 years [30]. In Drehlich et al.’s [30] study, adolescents found the Fitbit Flex easy to use, but maintenance requirements, such as charging and syncing the device, were burdensome [30]. Adolescents found the wearable useful for monitoring their PA and motivating them to be physically active [30]. This highlights TAM’s use for anticipating wearable acceptance (e.g., charging and syncing problems), which may be beneficial for the development of future wearable interventions. However, this study does not include the family nor all components of the TAM (external variables, attitudes, intentions to use, use).

The integration of multiple theoretical frameworks for understanding behaviour change has been documented [31]. The TDF [25] is an integrative framework synthesising key theoretical constructs used in behaviour-change theories to inform intervention implementation [32,33]. Behaviour-change experts identified, simplified, and evaluated existing behaviour-change theories to develop and validate a set of domains that explain behaviour change, resulting in the TDF [25,32]. The most recent and validated version of the TDF includes 14 domains: knowledge; skills; memory, attention, and decision processes; behavioural regulation; social/professional role and identity; beliefs about capabilities; optimism; beliefs about consequences; intentions; goals; reinforcement; emotion; environmental context and resources; and social influences [32]. The TDF forms part of the Behaviour-Change Wheel (BCW; [33]) and COM-B model [33]. The BCW is an integrative framework of 19 behaviour-change theories and enables interventions to be systematically developed. The BCW has been used to increase walking in adolescents and their parents [34], reduce sitting time in adults [35,36], and develop a PA app [37]. The TDF aligns with components of the COM-B model to explain an individual’s capability, opportunity, and motivation to participate in a behaviour [33]. The use of the TDF thus enables researchers to identify barriers and facilitators of behaviour change and map these onto intervention functions and BCTs that are anticipated to overcome identified barriers (using the BCW).

By utilising the TAM and TDF in this study, facilitators and barriers of wearable acceptance, wearable use, and PA can be anticipated and systematically targeted to inform future family-based wearable interventions.

### 1.3. Mixed-Methods Research

It is recommended that acceptability studies utilise both quantitative and qualitative methods (e.g., using mixed methods) [21]. Taking a mixed-methods approach enables researchers to understand the context and mechanisms of action (e.g., “why”) behind intervention/study effects [38]. A systematic review has highlighted wearable’s mixed acceptability, feasibility, and effectiveness for increasing child PA [13] but does not consider how the components may interact by integrating data [13,30]. Indeed, the importance of mixed-methods research is acknowledged [39,40], but the integration of quantitative and qualitative data are often undermined (e.g., presenting findings separately) [40,41]. This study utilised the Pillar Integration Process (PIP [41]), which is a novel analytical technique consisting of four-stages (listing, matching, checking, and pillar building) to systematically integrate quantitative and qualitative findings. It is expected to maximise synthesis and transparency within and between studies by outlining stages of integration that can be replicated [41]. Given the infancy of research exploring the acceptability of wearables in families, the integration of mixed-method findings may offer an informative contribution to the field.

Consequently, this study investigated the acceptability of using wearables in a family setting and how families’ experiences align with components of the TAM and TDF, combining quantitative and qualitative data using Pillar Integration Process [41]. The findings from this study will inform the development of a family-based wearable intervention.

## 2. Methods

This was a mixed-methods, pre–post observational study designed to assess families’ acceptability of wearables (Fitbit Alta HR).

### 2.1. Ethics

This study was approved by Loughborough University Ethical Approvals (Human Participants) Sub-Committee (REF 2020-1347-2029). All participants provided informed consent (parents on behalf of children), and children provided written assent. Parents had to confirm their child (if under 13 years of age) would not use the Fitbit app or online dashboard alone or on their own devices, in line with Fitbit’s current privacy policy [42].

### 2.2. Recruitment and Eligibility

Families were recruited via (1) social media advertisements and (2) existing connections. Participants were recruited between December 2020 and April 2021. Families were eligible if they had: (1) at least one child, aged 5 to 9 years, living in the same household; (2) access to a smartphone or computer device with Bluetooth or a USB port; (3) access to the Internet/Wi-Fi; and (4) lived in Yorkshire (England). All family members living in the same household were invited to participate.

### 2.3. Measures and Procedure

**Figure 1:**
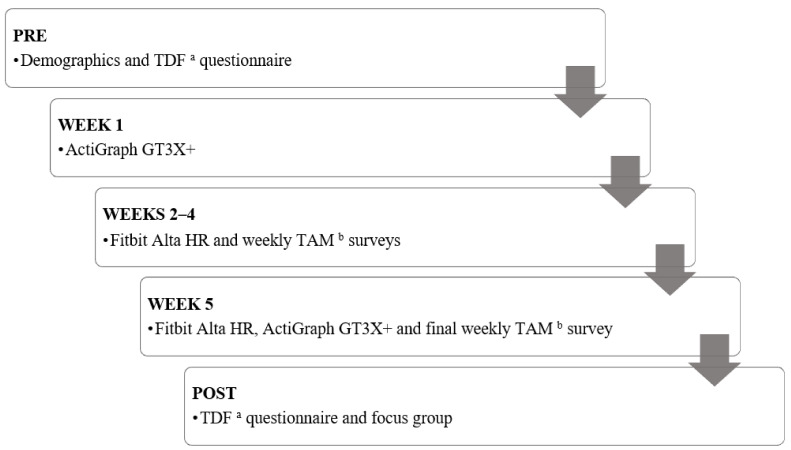
Study timeline. ^a^ TDF, Theoretical Domains Framework; ^b^ TAM, Technology Acceptance Model.

Figure 1 outlines the study timeline, and each measure is discussed below. Data collection took place between January and June 2021.

#### 2.3.1. Demographics

Age, sex (male, female, do not wish to say), ethnicity (open ended), home postcode (as an indication of deprivation, see Section 2.5.1), and ownership of a wearable (currently, previously, or never) were collected from all family members. Duration of wearable ownership (<1 month, 1–5 months, 6–11 months, 1–2 years, and <2 years) were collected from those who currently own a wearable. Adults’ highest educational qualification (none, General Certificate of Secondary Education (GCSE), Advanced level, National Vocational Qualification (NVQ) level 4, Bachelor’s degree, Master’s degree, doctorate (or equivalent for all options), or other) and employment status (full-time employed, part-time employed, self-employed, unemployed, full-time student, or other) were collected.

#### 2.3.2. The Theoretical Domains Framework (TDF) Questionnaire

Prior to and following Fitbit use, a parent/guardian completed the TDF questionnaire, which was used to assess beliefs of their own and their child’s (only those aged 5 to 9 years) capability, opportunity, and motivation to use a wearable and be physically active. The questionnaire (see Table S1) consisted of 14 questions, developed based on the COM-B model [33], and TDF [25,32]. Questions were mapped onto all components of the COM-B model (capability, opportunity, and motivation) and eight components of the TDF.

#### 2.3.3. The Technology Acceptance Model (TAM) Surveys

Weekly TAM surveys were used to assess the acceptability of using the wearable throughout the 4 weeks. The survey (see Table S2) consisted of eight (adult) to ten (child) statements and was adapted from a previous feasibility study [43]. Statements were aligned with components of the TAM [24], and participants responded on a 5-point Likert scale (Question 1: Poor, Fair, OK, Good, Excellent; Questions 2–10: Strongly disagree, Disagree, Neither disagree nor agree, Agree, Strongly agree). The surveys were parental report for children under the age of 10 years, but children aged ≥10 years were encouraged to complete the surveys themselves [44].

#### 2.3.4. Physical Activity

The ActiGraph GT3X+, a waist-worn tri-axial accelerometer [45], was used to measure PA. Devices were initialised using ActiLife v6 (ActiGraph, U.S) with a sampling rate of 60 Hz [46,47]. Prior to receiving the wearable and week 5 of the study (simultaneously with the Fitbit), all family members were instructed to wear the device on their right hip for seven consecutive days (24 h/day) and only to remove the device during water-based activities.

#### 2.3.5. Family Focus Groups

Families took part in a semi-structured telephone focus group up to two weeks after the study. Focus groups were conducted, recorded, and transcribed (by A.V.C). Questions were aligned to components of the TAM and TDF, following the same format as questions used in the TDF questionnaire and weekly TAM surveys (see Tables S1 and S2). Recommendations for conducting focus groups with children were considered, including using child-friendly language, regularly allowing the child(ren) to “pass” if they did not wish to answer a question, and enabling the child(ren) to provide answers before present adults to reduce the risk of coercion [48].

### 2.4. Materials

#### Wearable Activity Tracker

All family members received the Fitbit Alta HR for 4 weeks. The Fitbit was used as an acceptability/feasibility tool and did not contribute to the PA measurement (the ActiGraph GT3X+ was the only measure of PA in this study). The Fitbit Alta HR is a wrist-worn MEMS tri-axial accelerometer and optical heart rate (HR) tracker, with an OLED tap display [49]. The Fitbit’s default settings (e.g., daily step goal of 10,000) were set for use, but families could change this if they wished. Fitbit Alta HR displays a user’s daily steps, distance travelled, calories burned and active minutes, and HR [49]. The device provides users with reminders to move and feedback (vibration) when PA goals are met [49]. The Fitbit can be connected to a user’s smart device via the Fitbit app or online dashboard, which allows users to track their long-term PA levels, take part in virtual challenges, receive virtual badges and trophies, share information, and track other health behaviours (e.g., sleep) [49]. A researcher (A.V.C) set up each Fitbit device using a research-specific email address and password, which were provided to families and enabled access to the Fitbit app/dashboard. Family members were provided with a researcher-developed user manual instructing them how to set up, charge, and sync the Fitbit, but they were not instructed what features to engage with. The researcher manually connected all Fitbits to Fitabase, a data management platform used to support research projects using Fitbit and Garmin devices [50]. 

### 2.5. Data Analyses

#### 2.5.1. Demographic Data

Demographic data (*n*, %) are presented descriptively. Home postcode was used an indication of socio-economic status, using the Index of Multiple Deprivation (IMD) deciles (1–10) (England) [51]. Each neighbourhood in England is ranked from the 10% most deprived areas (decile 1) to the 10% least deprived areas (decile 10) [51]. Based on previous literature [52], postcodes in deciles 1–3 were considered “most deprived” and deciles 8–10 were considered “least deprived”.

#### 2.5.2. Fitbit Wear Time

A.V.C extracted step-count data from Fitabase [50] and determined the percentage of time family members wore the Fitbit. A step count of ≥1000 steps/day was used to determine a valid day as used previously [53,54]. The average number of valid days (≥1000 steps/day) per week wearing the Fitbit was calculated as an indication of “wearable use”. This was stratified into “adults” (≥18 years), “target children” (5 to 9 years), and “siblings” (<5 years and >9 years).

#### 2.5.3. Physical Activity Data

ActiGraph GT3X+ data were analysed using ActiLife v6 (ActiGraph, Pensacola, FL, USA) and downloaded in 60 s epochs. For children (<18 years), sleep was removed using the validated Sadeh [55] and Tudor-Locke’s [56] sleep-period-detection algorithms (which requires 60 s epochs). To determine PA intensities, data were summarised using the Freedson adult [57], Evenson et al. [58], and Pate et al. [59] cut points for adults, children aged 5 to 17 years, and younger siblings (<5 years), respectively. Children’s data were redownloaded into 15-second epochs, and adults’ data remained at 60 s epochs. These epoch lengths were selected since they correspond with the epoch lengths used to calibrate and validate the utilised cut-points as recommended [60]. Non-wear time was defined as ≥60 min, ≥20 min, and ≥10 min of consecutive zero counts for adult, children (5 to 17 years), and younger siblings (<5 years), respectively. A valid wear time was classified as ≥10 h/day for any four days for all age groups [61]. Given this study was an acceptability study, PA data were used to compliment qualitative findings and are presented descriptively in relation to the proportion of participants meeting the UK’s Chief Medical Officers’ PA recommendations of an average 60 min of MVPA per day (3 to 18 years) and an average of 150 min of MPA or 75 min of VPA per week (≥18 years). Results are stratified into “adults” (≥18 years), “target children” (5 to 9 years), and “siblings” (<5 years and >9 years).

#### 2.5.4. Thematic Analysis

A thematic analysis, utilising stages outlined by Braun and Clarke [62], was conducted on qualitative data using NVivo software (QSR International, Melbourne, Australia). After the familiarisation stage, two authors (A.V.C. and J.H.) independently developed “free codes” (initial codes), using an inductive approach, by coding each transcript line according to its meaning and content. Both authors independently developed free codes into themes using a deductive approach by grouping similar codes together and aligning them to components of the TDF [25,32]. Authors discussed each theme and refined them to create the final thematic themes, which included reviewing, defining, and naming themes [62]. Themes were not aligned with components of the TAM [24] during the thematic analysis (but were during the PIP [41]). Once themes were finalised, a third author (S.Co.), who has experience in thematic analysis, reviewed each theme alongside five transcripts (26%) and was encouraged to critically evaluate the themes produced. A.V.C., J.H., and S.Co. collectively refined final themes. This process has been used in similar studies to improve credibility [43]. Efforts were made to demonstrate rigour and trustworthiness using the “Big Tent Criteria” [63], such as authors (e.g., A.V.C.) demonstrating self-reflexivity by keeping field notes throughout the study and considering their own subjective biases. This included recognising their experiences using wearables for personal use and not transferring these preconceptions into the study’s focus groups.

#### 2.5.5. Pillar Integration Process (PIP)

**Figure 2:**
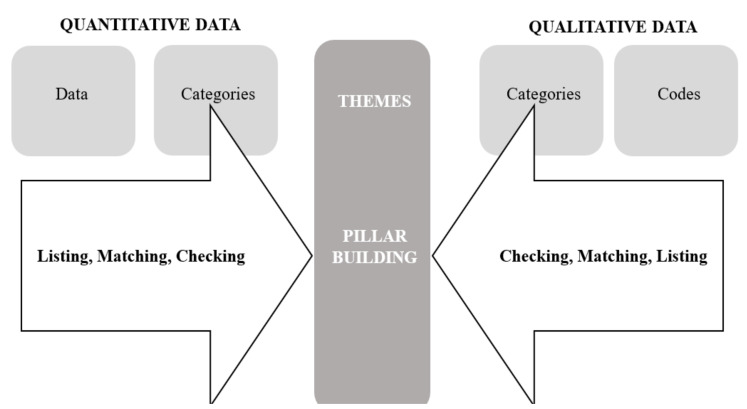
Pillar Integration Process [41].

Quantitative and qualitative findings were aligned using the four stages of the Pillar Integration Process (PIP): listing, matching, checking, and pillar building [41]. The method used is outlined in Figure 2, with a worked example (taken from Johnson et al. [41]) presented in Table 1. To conduct the PIP, A.V.C. listed quantitative findings and developed quantitative categories by summarising each quantitative finding (data) (listing). Quantitative categories were then matched with themes identified in the thematic analysis (which are referred to as “qualitative categories” using the PIP) (matching). A table was produced displaying how quantitative and qualitative categories aligned. Where categories did not align with a corresponding category, this was indicated (e.g., “no corresponding quantitative data”). This matching process was independently reviewed by J.H., who provided insight into where changes could be made (checking). The final stage involved “comparing and contrasting the findings, conceptualising the insights identified from connecting and integrating data and building inferences about what patterns, insights and themes have emerged and possible explanations” to build “pillars” [41] (pillar building). This stage was conducted by A.V.C., who aligned each row with the study aims. Pillars were derived by considering how the quantitative data aligned or contrasted with qualitative categories (thematic themes). Pillars were aligned with components of the TDF, which included considering whether the TDF component used to explain the qualitative categories (thematic themes) was still appropriate when paired with quantitative categories. This resulted in some pillars including more than one TDF component. Once pillars were developed, they were then organised according to components of the TAM, which reflect their overarching concepts (external variables, ease of use, usefulness, attitudes, and intentions). Pillars, including their alignments with TDF and TAM components, were checked by J.H., where necessary changes were discussed and made. The PIP results are displayed using tables (such as the example presented in Table 1); however, qualitative codes (participant quotations) are displayed in text.

## 3. Results

### 3.1. Recruitment and Retention

Thirty-eight parents/guardians expressed interest in the study, with twenty-four (63%) taking part. Of the 14 families that did not take part, most did not respond after expressing interest (*n =* 7, 50%). Two (12.5%) did not meet the eligibility criteria. Of the 24 participating families, one family (mother and 8-year-old daughter) returned the Fitbit after 5 days due to technical difficulties setting up the devices. They did not complete any further measures, but the mother took part in a one-to-one interview to discuss their reasons for discontinuing Fitbit use.

The number of participants providing valid ActiGraph data at baseline and week 5 were 20 (55.6%), 12 (41.4%), and 5 (41.7%) for adults, target children, and siblings, respectively. Response rates for the weekly TAM survey and the TDF questionnaire ranged from 92% to 100%.

Nineteen families took part in the focus groups, which lasted an average of 47 min (range: 35 min to 1 h). In 79% of cases (*n =* 15), all participating family members took part in the focus groups (range: 2–5). The remaining included only adult participants, with two including only one parent (1:1 interviews).

### 3.2. Demographics 

Table 2 includes demographic data for the 24 participating families (total 77 participants). Most families had two participating members (*n =* 8, 33%) (range: 2–5). Participants included 22 mothers, 11 fathers, 1 stepmother, 1 stepfather, 29 target children (5 to 9 years), 10 siblings, 2 cousins, and 1 grandmother.

### 3.3. Thematic Analysis

The thematic analysis resulted in 21 themes, which are displayed in Table 3. Thematic themes aligned with nine TDF components. Thematic themes were aligned with quantitative data as part of the Pillar Integration Process (PIP; [41]) and are included in the relevant pillar sections (Section 3.4) and tables (Table 4, Table 5, Table 6, Table 7, Table 8 and Table 9) under “qualitative categories”.

### 3.4. Pillar Integration Process (PIP)

The key findings from the TDF questionnaires and weekly TAM surveys are presented in the relevant pillar sections (under quantitative data and categories), with all findings presented in Tables S3–S5. Thematic themes were aligned with quantitative data using PIP, resulting in nine pillars. Pillars included 12 TDF domains (including skills, optimism, and intentions, which were part of the quantitative or qualitative categories but did not reflect the pillars overarching domain). Each pillar and how they align with components of the TAM (e.g., external variables, ease of use, etc.) are discussed below. How each pillar relates to components of the TDF are represented in brackets.

#### 3.4.1. External Variables

Table 4 displays the PIP used to explain external variables’ impact on PA and Fitbit use (Pillars 1 and 2).

**Pillar 1. The Fitbit’s impact on physical activity may be influenced by the family member’s pre-Fitbit physical activity levels (identity)**

Pillar 1 incorporated three quantitative categories and one qualitative category (thematic theme 1). The TDF component “identity” reflected the overall findings from Pillar 1 but also included “skills” and “beliefs about consequences” to reflect the quantitative data obtained from families (pre–post TDF questionnaires). All parents were confident their child had the physical abilities to be active, and most were confident they had the physical abilities to support their child’s PA (skills: an ability or proficiency acquired through practice). Most parents believed PA had a large impact on a child’s health and development (beliefs about consequences) and recognised its importance for their own mental health and well-being (including some children themselves):

*“When* *I was a bit stressed… Let’s go for a walk and let’s count how many steps we can get on this”*

(Mother, family 21).

*“When I was going outside it gave me a bit of nice nature feeling and that makes me feel calmer”*

(Female, 7 years, family 4).

Despite recognising the importance of PA, PA levels differed between family members. Approximately half of all family members were meeting PA guidelines before using the Fitbit (Table 4). Some families reported the Fitbit did not increase their PA level, as they were already active:

*“I do quite a lot of activity in my job anyway, so I know that I do a lot of steps”*

(Father, family 10).

*“Because I’m active I didn’t get more active but I decided not to do anything different”*

(Female, 8 years, family 19).

*“No because I did like, because I walk to school so I don’t, it was like I’d be doing it anyways”*

(Female, 12 years, family 16).

However, some families mentioned the Fitbit encouraged them to be more active despite already being active:

*“He [5-year-old child] has wanted to do more, even though he does enough…he likes to be physical anyways but looking at the Fitbit definitely helped”*

(Mother, family 2).

*“Normally we go out on the weekend anyway but this was another pusher for us to go out and get our 10,000 steps”*

(Mother, family 17).

**Pillar 2. Lack of time, school, work, and COVID-19 restrictions were barriers of Fitbit use and physical activity (environmental context and resources)**

Pillar 2 incorporated one quantitative category and two qualitative categories (thematic themes 2 and 3). The TDF component “environmental context and resources” reflected the findings from Pillar 2. Most parents were confident their child had the facilities, enough space, and, to a lesser extent, enough time to be active (environmental context and resources). This was supported by the fact no families mentioned facilities and space as barriers in the focus groups, but some referred to lack of time as a barrier to PA. Work and school were also considered barriers to being active and responding the Fitbit’s “reminders to move”:

*“Yeah but I just had to like to do it a bit secretly [steps] because I wasn’t allowed to move [at school]”*

(Female, 6 years, family 20).

*“I don’t think I have the scope because I work, I do desk work all day”*

(Father, family 16).

*“It really did depend if I was in class or not [if they responded to “reminders to move”] because I couldn’t just run out of class to get the steps… sometimes if fit into my break so that would be good”*

(Male, 9 years, family 3).

*“I feel like the only barrier to me is education because I’m having to do school sat down and then obviously after that I’ve also got to do revision”*

(Female, 17 years, family 8).

However, home-schooling and working from home, due to COVID-19 restrictions, were also considered barriers to PA. Despite most parents being confident their child had the facilities and space to be active, the inability to travel and closure of sports facilities (gyms and sports clubs) were considered barriers of parent and child PA:

*“I used to do jujitsu so that obviously, the restrictions that got cancelled… if I go to jujitsu, most likely I would have done a lot more steps”*

(Mother, family 8).

*“My issue was being sort of throughout the lockdown and home-schooling and the fact that I work from home as well, I don’t have time to–to do much physical activity”*

(Mother, family 5).

Positively, there was an increase in the number of parents who were confident their child has enough time to be physically active after using the Fitbit, with some parents reporting “making time” to be active (e.g., on weekends or using school breaktimes to be active):

*“You’ve got to make time to do something otherwise you just so busy”*

(Mother, family 21).

For some parents, work was considered a facilitator of PA (e.g., Pillar 1, having an active job).

#### 3.4.2. Wearable Use

Table 5 displays the PIP used to build Pillars 3 and 4, which reflect families’ use of the Fitbit.

**Pillar 3. The extent of Fitbit use varied throughout the study and between families (behavioural regulation)**

Pillar 3 incorporated one quantitative category and two qualitative categories (thematic themes 4 and 21) and reflects “behavioural regulation” and “decision processes”. Fitbit use was generally high (average of 71–91% throughout the study), but overall use ranged between 10% and 100%. In the focus groups, some families reported using the Fitbit daily, whereas others reported removing the device due to usability issues (Pillar 5) or forgetting to wear the device:

*“We wore them day in day out… I want to give it my best but my husband he was so diligent and having it on and making sure that he wore it and took every effort”*

(Mother, family 9).

*“I didn’t like looking at it at all, so I wore it, but I didn’t get anything from it”*

(Father, family 20).

*“I forgot a few times to put it on”*

(Male, 14 years, family 8).

Most families (*n =* 14) used step count, followed by heart rate (*n =* 9), and most reported using the Fitbit app (*n =* 11), mainly to monitor their own or their child’s (without child involvement) sleep (*n =* 9) (decision processes):

*“I liked uh I liked going on walks because you could check how much steps you’ve done”*

(Male, 10 years, family 15).

*“I found out that when I ran more it makes my heart go up really high and when I’m just like sitting down, erm, sitting down just like looking at something or watching something or doing something erm it would go down, so I really liked doing that, that was nice”*

(Female, 7 years, family 10).

*“Things that I haven’t considered before like the sleep states were the most interesting”*

(Father, family 16).

*“I didn’t really like the sleep bit in it because of that…Because they [parents] could check when I was sleeping”*

(Female, 12 years, family 16).

**Table 4:** The Pillar Integration Process used to build pillars reflecting external variables that impacted families’ PA and Fitbit use.

Quantitative Data (Source)	Quantitative Categories	Pillar (TDF Component)	Qualitative Categories (Themes Derived from the Thematic Analysis)
**Meeting PA guidelines (Pre-Fitbit ActiGraph data):** Adults: 55% Target children: 50% Siblings: 40%	Approximately half of all family members met PA guidelines before using the Fitbit.	**Pillar 1. The Fitbit’s impact on physical activity may be influenced by family member’s pre-Fitbit physical activity levels (identity)**	**Identity:** The Fitbit’s impact on physical activity is influenced by family member’s pre-Fitbit physical activity levels.
**Skills (TDF questionnaire)** All parents were confident their child had the physical abilities to be active and most believed they had the physical abilities to support their child’s PA (pre: 96%, post: 91%)	Physical abilities to be active was not a barrier to PA in this sample.		No corresponding qualitative categories
**Beliefs about consequences (TDF questionnaire)** 63–95% of parents reported PA had a large impact on a child’s physical health, mental health, social development, and, to a lesser extent, academic attainment (42–50%).	Parents recognised the importance of PA for child health and development but, to a lesser extent, academic attainment.		No themes developed using the thematic analysis reflected this finding, but some families mentioned the importance of PA for mental health/reducing stress.
**Environmental context and resources (TDF questionnaire)** 84–93% of parents were confident their child had the facilities and enough space to be active. Most (but fewer) parents were confident their child had enough time to be active (pre: 76%, post: 89%).	Having facilities and enough space to be active were not considered barriers of child PA. Fewer parents were confident their child had enough time to be active, but this increased from pre- to post-Fitbit.	**Pillar 2. Lack of time, school, work, and COVID-19 restrictions were barriers of Fitbit use and physical activity (environmental context and resources).**	**Environmental context and resources:** School and work as barriers of Fitbit use and physical activity
No corresponding quantitative data	n/a		**Environmental context and resources:** COVID-19 restrictions as a barrier of physical activity

Only one parent used the Fitbit app for PA-related data, which was exploring their heart rate in more detail:

*“It was really interesting to see that heart rate breakdown which I found was really good with the Fitbit app”*

(Father, family 15).

Inconvenience was the main reason for not using the app:

*“We all had varying levels of engagement with the app… Just the time and convenience of going on the app”*

(Step-mother, family 15).

Some parents (*n =* 5) reported that their child had lost interest in the Fitbit during the study or expected they would if they used the device long term. This is reflected in the quantitative data, where adults and siblings used the Fitbit less in the final week and target children in week 3 (see Table 5):

*“I already saw by the end of week 4 the novelty was wearing off”*

(Mother, family 3).

*“I didn’t know it’d be on for that long”*

(Male, 10 years, family 3).

*“* *Like anything new you’re watching it advertently for the first couple of weeks then you just wear it, I don’t know if you then go back to the app on a regular basis. I don’t particularly do that”*

(Father, family 4).

**Pillar 4. Individual and collective Fitbit use (social influences)**

Pillar 4 incorporated one quantitative category and one qualitative category (thematic theme 7) and reflected the TDF component “social influences”. Most parents were confident their child had someone to be active with although the number reduced after using the Fitbits (social influences). Some families reported using the Fitbit collectively (*n =* 3), such as obtaining steps by going on walks together:

*“We [self and 8-year-old daughter] were quite excited you know in the evenings… lets go for a walk and lets count how many steps we can do on this”*

(Mother, family 21).

Most families reported comparing PA levels and/or competing against one another (Pillar 6). However, some parents reported a lack of “collective experience” due to the inability to sync all family members Fitbit’s to one smart device/app:

*“We didn’t really do anything together”*

(Father, family 6).

*“We couldn’t link our accounts as a family, which sort of ruins that experience as a family because it didn’t—that sort of collective didn’t exist”*

(Step-mother, family 15).

*“Because they were on different devices there were no direct comparison between as a family”*

(Father, family 4).

Some family members used the Fitbits alongside individuals not involved in the study, such as peers and extended family members:

*“I run around the park and in my Fitbit with grandma and grandad”*

(Female, 10 years, family 10).

*“My friend at school…right, we’ll try and get to a thousand on my Fitbit and then we’d run around the ball court”*

(Female, 7 years, family 10).

#### 3.4.3. Ease of Use

Table 6 displays the PIP used to build Pillar 5, which reflects the Fitbit’s ease of use.

**Pillar 5. Family members found the Fitbit easy to use but reported issues with usability (emotion) and difficulties interpreting Fitbit outputs (knowledge)**

**Table 5:** The Pillar Integration Process used to build pillars reflecting families Fitbit use.

Quantitative Data (Source)	Quantitative Categories	Pillar (TDF Component)	Qualitative Categories (Themes Derived from the Thematic Analysis)
**Wearable use (Fitabase data)** **Week 1:** Adults: 87% Target children: 81% Siblings: 81% **Week 2:** Adults: 85% Target children: 91% Siblings: 78% **Week 3:** Adults: 86% Target children: 71% Siblings: 81% **Week 4:** Adults: 73% Target children: 74% Siblings: 75%	Large variation in Fitbit use throughout the study, with families using the Fitbit the least in the final week (week 4) and target children reducing their use in week 3 and 4.	**Pillar 3. The extent of Fitbit use varied throughout the study and between families (behavioural regulation)**	**Behavioural regulation:** The extent of Fitbit use.
No corresponding quantitative data	n/a		**Decision processes:** Use of the Fitbit’s features and partnering application.
**Social influences (TDF questionnaire)** 85–97% of parents were confident their child has someone to be active with (pre: 97%, post: 85%).	Most parents reported their children had someone to be active with, but this number reduced after using the Fitbit.	**Pillar 4. Individual and collective Fitbit use (social influences)**	**Social influences:** Individual and collective Fitbit use

Pillar 5 incorporated two quantitative categories and two qualitative categories (thematic themes 10 and 12) and reflected TDF components “emotion” and “knowledge”. Most family members found the Fitbit easy to use:

*“I think the app was really kid friendly”*

(Father, family 4).

*“It was really easy to set up as well and like it just works really well… all I had to do were charge it up once a week and take it off when you had a bath that was it”*

(Father, family 6).

However, the number of family members reporting problems with the Fitbit ranged from 7% to 64%. Some family members reported the Fitbit Alta HR was easier to use than wearable devices they currently use or had previously used, whereas others reported their own wearable was easier to use:

*“I found that in general um it was a little bit more user-friendly than the Garmin [own wearable] I found that the things were more uh entry level based”*

(Step-mother, family 15).

*“I’d probably say the Charge 3 [own wearable] just because it was a lot easier to use, than the Alta one”*

(Mother, family 3).

Families reported difficulties charging, syncing, or setting up the devices (*n =* 6), and one parent decided to withdraw from the study due to difficulties with Fitbit set up (emotion). Nine families reported the Fitbit screen was unresponsive, and the inability to link multiple Fitbit’s on to one smart device/app (e.g., Pillar 4) was noted as a usability issue:

*“You had to press quite ferociously for it to either swap slides or for it to turn back on and it’s quite annoying do that every single time you wanted to see the time or the steps”*

(Father, family 20).

*“I did struggle sometimes putting it on to charge and I don’t know if that was me or I don’t know”*

(Mother, family 13).

*“The standout reason [for withdrawing from the study] was it was too much time and energy and effort to actually register and get it started in the first place”*

(Mother, family 14).

Eleven families found some Fitbit outputs difficult to interpret, including distance travelled and calories (children), symbols (“fire” and “light bulb”) (children), and active minutes (parents). Some parents and one child reported they (or their child) were too young to understand some of the Fitbit’s outputs (knowledge). This may contribute to why fewer parents reported that target children found the wearable easy to use:

*“You know the one with like the fire symbol on, I don’t know what that was”*

(Female, 12 years, family 16).

*“The thing how it says how much calories you’ve burnt. I don’t understand how we could not go outside for a day and still have burnt a thousand calories”*

(Male, 9 years, family 6).

*“I don’t really understand the calories that I’ve burnt. But when I’m older I’ll probably understand more*”

(Male, 10 years, family 17).

*“I just think it’s a bit of a mystery… it just appears as a number of minutes”*

(Father, family 4).

**Table 6:** The Pillar Integration Process used to build pillars reflecting the Fitbit’s ease of use.

Quantitative Data (Source)	Quantitative Categories	Pillar (TDF Component)	Qualitative Categories (Themes Derived from the Thematic Analysis)
**Easy to use (TAM weekly surveys)** 61–91% of participants agreed the Fitbit was easy to use. Adults: 79–85% Target children: 61–78% Siblings: 64–91%	Most family members found the Fitbit easy to use, with fewer parents reporting their children (target child; 5 to 9 years) found the Fitbit easy to use.	**Pillar 5. Family members found the Fitbit easy to use but reported issues with usability (emotion) and difficulties interpreting Fitbit outputs (knowledge)**	**Emotion:** Fitbit usability
**Problems using (TAM weekly surveys)** 7–64% of participants reported experiencing problems with the Fitbit. Adults: 21–30% Target children: 7–26% Siblings: 25–64%	Some problems with the Fitbit were experienced, which were mainly experienced by siblings (but large variations in all age groups)		
No corresponding quantitative data	n/a		**Knowledge:** Interpretation of Fitbit outputs

#### 3.4.4. Usefulness

Table 7 displays the PIP used to build Pillars 6 and 7, which reflect the Fitbit’s usefulness for increasing PA and other-related behaviours.

**Pillar 6. The influence the Fitbit has on physical activity and health behaviours varied amongst family members (beliefs about consequences)**

Pillar 6 is presented in two parts: (1) the Fitbit’s impact on PA and health behaviours and (2) the mechanisms of action behind the Fitbit’s impact on PA. Pillar 6 incorporated five quantitative categories and five qualitative categories (thematic themes 5, 8, 16, 17, 19). The former part reflects the TDF components “beliefs about consequences” and “optimism”, with the latter also reflecting “social influences”, “behavioural regulation”, and “reinforcement:” depending on the mechanism of action (as indicated below).

**The Fitbit’s impact on PA and health behaviours:** The ActiGraph data demonstrated a slight increase in the number of family members meeting PA guidelines after using the Fitbit (Table 7). The number of parents reporting they were more active as a family because of the Fitbit increased over the study. On an individual level, most parents believed the Fitbit increased their child’s PA levels, with the number of parents who were confident the Fitbit could increase their child’s PA levels almost doubling after using the Fitbit (optimism: the confidence that things will happen for the best or that desired goals will be attained). Fewer parents reported the Fitbit increased their own PA levels and motivation to be active, with some suggesting that external factors (Pillars 1 and 2) or having already used a wearable contributed to this:

*“It increased my physical activity. I wanted to achieve my 10,000 steps a day”*

(Male, 11 years, family 17).

*“It changed how much I wanted to go outside to get my* *steps—to achieve things”*

(Male, 9 years, family 6).

*“I’m one of those people I* *think I need somebody really cracking the whip. It didn’t make a huge difference as sort of making me want to be more active”*

(Mother, family 9).

*“I found it fairly motivational but that was slightly offset by the fact that I’ve already got a fitness tracker anyway, so it’s not a novelty to start that where someone might* *get a Fitbit that hasn’t had that before, they might get that initial motivation”*

(Father, family 4).

*“I didn’t change anything. I didn’t put it on and think oh now I’ve got this on, I’m going to go on a walk every day”*

(Mother, family 3).

Two parents reported that motivation needed to come from “within” (intrinsic motivation). Some family members also reported “trying to get more sleep” or a change in diet because of the Fitbit; e.g., one parent reported adapting their calorie intake based on their calorie expenditure (monitored using the Fitbit):

*“When I burn my 2000 calories, yes I burned my 2000 calories so now I have to eat less”*

(Father, family 9).

*“With this [Fitbit], we were being careful about the eating”*

(Mother, family 21).

*“We were saying to her that you need to sleep more hours coz she’s too busy reading on a night instead of going to sleep and if you check on the app, we kept synching it and checking on the app to see if her sleep improved”*

(Mother, family 10).

**The mechanisms of action behind the Fitbit’s impact on PA:** Three mechanisms of action behind the Fitbit’s impact on PA were identified in the qualitative data and are discussed below.

**Competition and comparison (social influences):** Most family members reported comparing PA levels (*n =* 12) and competing with one another (*n =* 9). Comparison and competition typically referred to step count or reaching a step goal. Four families mentioned the negative impact of comparing and competing, such as children feeling upset:

*“Well I enjoyed having the competition against my brother because me and my brother are always trying to beat each other with the amount of steps and get 10,000 first”*

(Female, 10 years, family 7).

“Well I didn’t really want to compare them because I thought they might be a bit more than me … I was going to do a thing where at the end of the day there’s a winner for how many—for the biggest amount of steps, but I quitted that because I saw mum did a lot of steps at work… it made me jealous”

(Female, 7 years, family 4).

**Monitoring and goal setting (behavioural regulation):** Most families (*n =* 11) reported monitoring their PA levels and working towards PA goals. Eleven families reported goal setting encouraged them to be active. Typically, families referred to reaching “10,000 steps a day”. Fewer families referred to smaller (hourly) goals or goals that did not refer to step count. Three children increased their goal (15,000–20,000 steps/day), and one reduced their goal (6000 steps/day). Two parents referred to “active minutes” and “calories” as their daily goal instead of step count:

*“She [child] was running round the kitchen more because she wanted to get up to the next kind of level of 100 steps”*

(Mother, family 4).

*“I liked if you met your targets or not, like you’re 1 out of 5 [active days] and 250 steps an hour, it would tell you if you hit that goal for that hour”*

(Mother, family 13).

*“Sometimes if I’d already got 10,000 steps in the morning, I’d go for like 28,000”*

(Male, 9 years, family 3).

*“Yeah we lowered it to 6000… it made me feel a bit easier…I got calmer and calmer and did normal things and got 14,000 steps” (*

Female, 7 years, family 4).

**Prompts and reinforcement (reinforcement):** Fewer families mentioned reinforcement (*n =* 8) and prompts (*n =* 3) as ways the Fitbit encouraged them to be active. Some found the hourly “reminders to move” motivating, whereas other family members did not and reported being unable to respond to them due to external factors (e.g., Pillar 2). Some children used the Fitbit’s feedback (vibration and virtual fireworks) as motivation to reach their PA goal. Children from two families enjoyed receiving the Fitbit’s virtual badges, and two parents reported the Fitbit’s auto-recognition they were being active (via a vibration) was motivating:

*“I didn’t want to do it [respond to prompts] because I was too relaxed in the chair”*

(Female, 9 years, family 20).

*“You obviously get the buzz when you hit 10,000 steps but also it does it when it when you’re doing a workout or something like that and I, I quite like that as a sort of motivation technique”*

(Mother, family 16).

*“I like the firework when you get to 10,000”*

(Male, 6 years, family 1).

**Pillar 7. The Fitbit increased awareness of physical activity levels, but some families were unsure how this reflected physical activity guidelines (knowledge)**

Pillar 7 incorporated four quantitative categories and two qualitative categories (thematic themes 13 and 14). The TDF component “knowledge” reflected the overall findings from Pillar 7 but also included “beliefs about consequences”. After using the Fitbit, all parents believed it would be beneficial to learn more about their child’s PA levels (beliefs about consequences). Parents reported being more aware of their own and their child’s PA levels. The number of parents who were confident their child was meeting PA guidelines increased after using the Fitbit, with some parents referring to whether their child was meeting their goal of 10,000 steps a day and others referring to how active their child was compared to them (based on step count):

*“I would probably say that [child] was overachieving and I was underachieving …one night he’d done 24,000 steps and I thought how has he done that?!”*

(Mother, family 13).

*“I thought that I’d probably do…burn more calories because I’m always on my feet so I used to say oh I never sit down and I must burn so much calories so I think it made me understand that I didn’t burn as much as I thought I did”*

(Mother, family 10).

*“I like looking at the miles because I’ve never really known like how many miles I’ve done in a day”*

(Father, family 1).

Despite this, the number of parents reporting they had a lot of understanding: of the term MVPA decreased after using the Fitbit. Some families (*n =* 6) stated they were unsure of PA recommendations and questioned whether (and how) step count (*n =* 4), heart rate (*n =* 2), and active minutes (*n =* 1) were equivalent to government guidelines. Two parents stated the Fitbit decreased their understanding, due to the differences in terminology (“active minutes” vs. “MVPA”). On the other hand, the number of parents reporting they had “some understanding” of the term MVPA increased, but only one parent recalled the Fitbit “signposted” to information about the World Health Organisation’s PA recommendations:

*“I mean they could walk gently and do 10,000 steps; it showed how much he was doing. It didn’t—it didn’t teach me how much he should be doing”*

(Mother, family 7).

*“You know they reckon that the 10,000 steps had been plucked from nowhere and it might actually be less steps you have to do a day to count”*

(Mother, family 19).

*“* *I found that the Fitbit really acknowledged activity easily, erm which my understanding of moderate to vigorous exercise would really be quite a good raised heart rate, erm but I found on the Fitbit that it seemed to acknowledge activity just going out for walks”*

(Mother, family 4).

*“I know what moderate means but then vigorous I’ve got no idea what you’re trying to aim at”*

(Mother, family 8).

*“The World Health Organisation stuff that it brings up quite often, that was quite interesting”*

(Father, family 16).

**Table 7:** The Pillar Integration Process used to build pillars reflecting the Fitbit’s usefulness for increasing physical activity and other related behaviours.

Quantitative Data (Source)	Quantitative Categories	Pillar (TDF Component)	Qualitative Categories (Themes Derived from the Thematic Analysis)
**Changes in the number of participants meeting PA guidelines (ActiGraph data: pre➔post)** Adults: 55% ➔ 60% Target children: 50% ➔ 67% Siblings: 40% ➔ 60%	A slight increase in the number of participants meeting PA guidelines.	**Pillar 6. The influence the Fitbit has on physical activity and other health behaviours varied amongst family members (beliefs about consequences)**	**Beliefs about consequences:** The influence of the Fitbit on physical activity
**Fitbit’s impact on PA motivation (TAM weekly surveys)** 53–89% of participants reported the Fitbit motivated them to be more active dults: 53–62% Target children: 68–89% Siblings: 73–82%	The Fitbit motivated family members to be more active, especially children (vs. adults).		
**Fitbit’s impact on individual PA (TAM weekly surveys)** 50–81% of participants agreed they were more active because of the Fitbit. Adults: 50–53% Target children: 61–81% Siblings: 65–74%	Family members perceived an increase in PA because of the Fitbit, but this was less so for adults.		
**Fitbit’s impact on family PA (TAM weekly surveys)** The number of parents reporting they were more active as a family because of the Fitbit increased (week 1: 62%, week 4: 73%)	More reported participation in PA as a family as the study went on.		
**Optimism (TDF questionnaire)** Parent’s confidence the Fitbit could increase their child’s PA increased (pre: 48%, post: 81%)	Parents were more optimistic the Fitbit can increase their child’s PA levels after using the device for 4 weeks.		
No corresponding quantitative data	n/a		**Beliefs about consequences:** The Fitbit’s impact on health outcomes other than physical activity (e.g., sleep and diet)
No corresponding quantitative data	n/a		**Social influences:** Competition and comparison as mechanisms of action
No corresponding quantitative data	n/a		**Behavioural regulation:** Monitoring and goal setting as mechanisms of action
No corresponding quantitative data	n/a		**Reinforcement:** Prompts and reinforcement as mechanisms of action
**Knowledge (TDF questionnaire)** The number of parents reporting they had “a lot of understanding” of the term MVPA decreased (pre: 54%, post: 36%). The number reporting “some understanding” increased (pre: 21%, post: 41%).	Mixed opinions regarding perceived knowledge of the term MVPA.	**Pillar 7. The Fitbit increased awareness of physical activity levels, but some families were unsure how this reflected physical activity guidelines (knowledge)**	**Knowledge:** The Fitbit’s (in)ability to improve understanding of physical activity guidelines
**Knowledge (TDF questionnaire)** 42–50% of parents accurately reported guidelines of 60 min of MVPA per day. Less parents reported PA guidelines of <60 min of MVPA per day after using the Fitbit (pre: 38%, post: 23%).	Increased understanding of child PA guidelines (duration).		
**Knowledge (TDF questionnaire)** The number of parents who were confident their child was meeting PA guidelines increased (pre: 65%, post: 92%). Post Fitbit, no parents reported they were not confident.	Increased confidence their child was meeting guidelines; however, most were confident before using the Fitbit.		**Knowledge:** The Fitbit increased awareness of physical activity levels
**Beliefs about consequences (TDF questionnaire)** Parents believed it would be beneficial to learn more about their child’s PA levels (pre: 93%, post: 100%).	Parents recognised the importance of learning about their child’s PA levels.		

#### 3.4.5. Attitudes

Table 8 displays the PIP used to build Pillar 8, which reflects families’ attitudes towards using the Fitbit.

**Pillar 8. Fitbit aesthetics and perceived accuracy impacts feelings towards using the Fitbit (emotion)**

Pillars 8 incorporated five quantitative categories and two qualitative categories (thematic themes 11 and 18). The TDF component “emotion” reflected the overall findings of Pillar 8 but also included “beliefs about consequences”. Most parents rated their family’s experience using the Fitbit as “Good”. Few family members were embarrassed to wear the Fitbit, and over half of all families enjoyed wearing the Fitbit, particularly target children. Few family members were embarrassed to wear the Fitbit; however, there was a large variation in the number of family members who found the Fitbit uncomfortable. Some family members reported the Fitbit strap was uncomfortable/tight or caused skin irritation. The number of parents who reported they expect a wearable to increase their stress levels increased after using the Fitbit, which may be due to the Fitbit’s usability (Pillar 5) or fears their child may lose or break the device:

*“It hurt and it—it made a mark on my arm”*

(Male, 5 years, family 15).

*“I was like let’s just put it all away because we’re not using it and I don’t want to break it”*

(Mother, family 14; withdrew from the study).

*“I found it quite uncomfortable”*

(Step-mother, family 15).

*“I think sometimes just my skin—I—it got like to the bottom of the Fitbit—just got a bit like some peeled off and it was irritating”*

(Male, 11 years, family 17).

*“Was a bit of a chaos and a bit stressful because we couldn’t—I think the main reason why we couldn’t identify which one was which”*

(Mother, family 17).

Stress and unenjoyment may also have been a result of the Fitbit’s perceived inaccuracy to capture PA. Members from seven families felt the Fitbit was not accurate, particularly that the Fitbit underestimated step count compared to their own wearable or monitoring (counting steps) (beliefs about consequences). Other family members reported the Fitbit was inaccurate at capturing heart rate and active minutes. One parent reported accuracy would determine their intention to use a wearable again (Pillar 9). Not only that, but some family members also reported the Fitbit’s inability to recognise certain activities (cycling and weightlifting) frustrating:

*“It had like 2 more steps to do so I did those two more steps and then noticed that actually it wasn’t picking it up”*

(Female, 10 years, family 7).

*“I wore them on the same hand [study and own device] 3 or 4% difference or something like that which was surprising*”

(Father, family 20).

*“Doing squats or lifting weights or whatever it may be, it didn’t seem to be able to recognise that as activity”*

(Father, family 6).

**Table 8:** The Pillar Integration Process used to build pillars reflecting families’ attitudes towards using the Fitbit.

Quantitative Data (Source)	Quantitative Categories	Pillar Building (TDF Component)	Qualitative Categories (Themes Derived from the Thematic Analysis)
**Overall experience (TAM weekly surveys)** Most parents rated their family’s experience using the Fitbit as “Good” (39–47%). No families reported their experience was “Poor”, and only 3–9% (week 1: 9% and week 4: 3%) reported their experience was “Fair”. “Excellent” responses increased from 25% (week 1) to 39% (week 4).	Most families had a good experience using the Fitbit, and the number of families reporting they had an excellent experience increased from week 1 to 4.	**Pillar 8. Fitbit aesthetics and perceived accuracy impacts feelings towards using the Fitbit (emotion)**	No corresponding qualitative categories
**Comfort (TAM weekly surveys)** 11–64% of participants agreed the Fitbit was uncomfortable to wear. Adults: 18–29% Target children: 11–30% Siblings: 25–64%	Large variation in the number of family members finding the Fitbit uncomfortable. More siblings found the Fitbit uncomfortable.		**Emotion:** Fitbit aesthetics impacts enjoyment of using the Fitbit
**Enjoyment (TAM weekly surveys)** 55–96% of participants liked wearing the Fitbit. Adults: 71–74% Target children: 86–96% Siblings: 55–73%	Family members liked wearing the Fitbit, particularly target children.		
**Embarrassment (TAM weekly surveys)** 0–4% of participants were embarrassed to wear the Fitbit.	Family members were not embarrassed to wear the Fitbit.		
**Emotion (TDF questionnaire)** The number of parents who reported they expect a wearable to increase their stress levels increased from using the Fitbit (pre: 29%, post: 36%). However, this decreased for children’s stress levels (pre: 24%, post: 19%).	Some family members reported the Fitbit increased their stress levels. Fewer parents expected the Fitbit to increase their stress levels than actually did after using the Fitbit (29% vs. 36%). Few families (but some) reported the Fitbit increased their child’s stress levels.		
No corresponding quantitative data	n/a		**Beliefs about consequences:** The Fitbit’s ability to capture physical activity

#### 3.4.6. Intentions

Table 9 displays the PIP used to build Pillar 9, which reflects families’ (1) intentions and considerations for future wearable use and (2) considerations for future wearable interventions (see Table 9).

**Pillar 9. Intentions and considerations for future wearable use and wearable interventions (decision processes)**

Pillar 9 incorporated two quantitative categories and four qualitative categories (thematic themes 6, 9, 15, and 20) and reflected “decision processes” as part of the “memory, attention, and decision processes” TDF domain and “intentions”. Considerations for future wearable interventions reflected domains “social influences”, “behavioural regulation”, and “knowledge”.

**Intentions and consideration for future wearable use:**

Most adults would consider purchasing a Fitbit or similar device for themselves and target children and were willing to incorporate a wearable into their child’s daily routine (intention: a conscious decision to perform a behaviour or a resolve to act in a certain way). However, most families (*n =* 13) reported wanting a more “feature-rich” device (e.g., link with other apps, answer phone calls), and for children, wearable aesthetics (e.g., colourful straps) and gamification components were important. Families reported being willing to use a device that was more comfortable, waterproof, or easier to use:

*“If there’s a kids range focused on kids that would encourage them to wear it more often as well and be a bit more interested in it as well if it was more focused on you know kids range of straps”*

(Father, family 4).

*“It would be waterproof”*

(Male, 9 years, family 6).

*“Well I would kind of like it to have a little game on it”*

(Female, 7 years, family 4).

*“I want one that’s a little bit more higher tech”*

(Father, family 1).

Reasons for not wanting to use a device again were due to the inconvenience of charging and syncing the device or their anticipated novelty effect. Two families referred to waiting until their child was older to purchase a device (5 years and 10 years):

*“No, I love the idea of it but no I just wear it to begin with then get fed up”*

(Grandmother, family 13).

*“I didn’t know whether she [7-year-old child] would have the novelty or if that would wear off* *“*

(Father, family 4).

*“* *I would use it again if obviously if it was comfortable* *”*

(Female, 17 years, family 8).

*“He’s only 5 if he was a little bit older, I would consider getting him one”*

(Mother, family 13).

*“If she does move less as she gets older then I’d maybe consider it”*

(Mother, family 19).

*“Maybe when I’m older, but I don’t think so right now”*

(Male, 10 years, family 17).

**Considerations for future wearable interventions:**

Suggestions for future wearable interventions were divided into three themes in the thematic analysis (but all form part of Pillar 9) and are discussed below.

**Competition (social influences):** Most families (*n =* 12) suggested incorporating competition into a future intervention, which included within- and between-family competitions. Providing rewards or incentives (badges, certificates) were mentioned by some children. Four families mentioned the potential negative impact of competition, with one parent suggesting the importance of making competitions optional:

*“You give the other families the Fitbits and like uh some—a few families the Fitbits and see who gets the most—add all their steps and see how—which people get the most steps”*

(Male, 11 years, family 17).

*“Instead of automatically getting, you know, roped into a competition, you would submit it willingly and say that we have this many steps or whatever, perhaps. But then again, it—if not—if people aren’t gonna be proud of what they’ve done that week or they sort of drop back then there’s not really a competition element to it”*

(Step-mother, family 15).

*“That would be kind of unfair because one family might have like ten children but then another family might have four”*

(Male, 11 years, family 17).

**Tailor towards the individual and their routine (behavioural regulation):** Four families mentioned the importance of tailoring a future wearable intervention to individuals and their routine. Some suggestions included focusing challenges on weekends, disabling prompts during work hours (e.g., Pillar 2), or using a wearable that recognised periods of inactivity and provided prompts during these time periods:

*“I think that comes down to the you know the routine you know if you were inactive on a Tuesday and a Thursday the Fitbit registers that pattern”*

(Father, family 1).

*“What I think would be really useful is for you to be able to put in your routine, so you could set things like don’t be vibrating when you’re in school or when you’re at work, but then make sure you prompt me in between these times cos that’s when I am able to do something and when I need encouragement that sort of thing”*

(Mother, family 3).

**Incorporate more PA information (knowledge):** Six families wanted more information about PA guidelines, such as the number of steps a child should take a day, with one parent suggesting a “rating scale” to indicate the intensity of the activity being carried out (e.g., 5 = moderate, 10 = vigorous). Some suggested that the Fitbit’s availability of features and instructions on how to use them needed explaining further:

*“Like children should be aiming to do however many steps a day or something like that”*

(Mother, family 12).

*“* *I don’t know if you could have more of a number scale or something like that, you know 1 to 10… 10 is vigorous you know, 5 is moderate, that type of thing”*

(Mother, family 10).

“Explain what active minutes means… is it the same as intensity minutes?”

(Mother, family 4).

**Table 9:** The Pillar Integration Process used to build pillars reflecting families’ intention to use a wearable in the future and considerations for future wearable use and wearable interventions.

Quantitative Data (Source)	Quantitative Categories	Pillar Building (TDF Component)	Qualitative Categories (Themes Derived from the Thematic Analysis)
**Purchasing a device (TAM weekly surveys)** 42–78% of participants would consider purchasing a similar device in the future Adults: 64–76% Target children: 71–78% Siblings: 42–67% (week 1: 67%, week 4: 42%).	Large variation. Fewer parents reported they would be willing to purchase a similar device for siblings, which decreased overtime. More willing to purchase device for target child.	**Pillar 9. Intentions and considerations for future wearable use and interventions (decision processes)**.	**Decision processes:** Intentions and considerations for future wearable use
**Intentions (TDF questionnaire)** Less parents reported they would be willing to incorporate more PA into their child’s daily routine (pre: 97%, post: 92%), but more reported they would be (very) willing to incorporate a wearable into their child’s daily routine (pre: 83%, post: 88%) after using the Fitbit.	A decrease in willingness to incorporate more PA into their child’s routine may be due to the increase in PA because of the Fitbit		
No corresponding quantitative data	n/a		**Suggestions for future interventions:** **Social influences:** Competition **Behaviour regulation:** Tailor towards the individual and their routine **Knowledge:** Incorporate more PA information

## 4. Discussion

The aim of this study was to explore the acceptability of families’ using wearables and how experiences align with components of the Technology Acceptance Model (TAM; [24]) and the Theoretical Domains Framework (TDF; [25,32]) using the Pillar Integration Process (PIP; [41]). Nine pillars were produced, aligning with all TAM domains and incorporating 12 TDF domains (nine overarching domains). This is one of the few studies to explore families’ acceptability of wearables [13] and the first to use the TDF and PIP to inform the findings. Fitbits were considered easy and enjoyable to use, but their perceived impact on PA were mixed, and external variables (e.g., work, school) may have contributed towards this. Positively, most parents were willing to purchase a wearable for themselves and children, demonstrating their acceptability.

The TAM [24] has previously been used to quantitatively explore the acceptance of wearables in adults [26,27,28,29]. These studies demonstrated that perceived ease of use and usefulness of wearables (e.g., “ability to accomplish more”) predicts wearable use [26,27], with perceived usefulness also predicting attitudes towards use [27]. One qualitative study previously used the TAM to understand wearable acceptance in adolescents, and the findings reflect those of this study (usability issues, mixed opinions as to whether the wearable increased PA levels) [30]. However, Drehlich et al.’s [30] study only explored perceived ease of use and usefulness in adolescents. The current study also explored external variables that may influence wearable use and PA, attitudes towards the Fitbit, intention to use wearables, and experiences using the Fitbit, which may provide valuable insights for developing family-based wearable interventions. The use of the TDF enabled facilitators and barriers of family wearable use and their impact on PA to be identified. For example, parents’ knowledge of PA guidelines (knowledge) and optimism that a Fitbit could increase their child’s PA levels (optimism) increased after using the Fitbit. When utilised alongside the COM-B model [33] these findings demonstrate Fitbit’s ability to overcome barriers of psychological capability and automatic motivation [33]. Key findings and their implications are discussed.

### 4.1. External Variables 

Families reported that already being active was one reason for the Fitbit not impacting their PA levels. This has also been identified in a systematic review that explored the acceptability, feasibility, and effectiveness of wearables for child and adolescent PA [13] although evidence is mixed. For example, Gaudet et al. [64] found that after using the Fitbit Charge HR for 7 weeks, adolescents participating in regular PA before the intervention increased their MVPA, whereas no changes in MVPA were seen in those that did not participate in regular PA or had no/little intention to do so [64]. This evidence suggests that wearables may be more effective for already active children, which is contradictory to the results found in this study. The impact of pre-wearable PA levels on changes in PA must be explored further and considered (e.g., controlled for) when implementing wearable interventions. Notably, work and school were identified as barriers of wearable use and being active, which may highlight the importance of involving the school and workplace alongside families in future wearable interventions. Some suggest that this integration across settings may increase intervention effectiveness [65], but school-based interventions that incorporate a wearable appear mixed in their effectiveness [13]. It is unclear the extent to which wearables have been incorporated into workplace interventions, but there is some evidence that wearable-based interventions can reduce workplace sedentary behaviour [66].

### 4.2. Wearable Use 

This study found that Fitbit use was relatively high, but there was an apparent decline in weeks three and four. Ridgers et al. [67] recently suggested an “adherence window” of 2–4 weeks, which may reflect a window of opportunity (e.g., [64,68,69]) for researchers to encourage long-term wearable use. However, it is still unclear how this can be achieved. One study focused on how older adults (≥65 years) formed a habit (“behavioural patterns enacted automatically in response to a situation in which the behaviour has been performed repeatedly and consistently in the past” [70], p. 2) of using their wearable (for at least 6 months, and devices were pre-owned, not used as part of a study or intervention) [70]. Techniques, such as graded task, contextual, and temporal cues and anticipating barriers of wearable use, were identified [70,71]. Thus, implementing these strategies into wearable interventions may increase older adults’ engagement with the wearable, but it is unclear whether this would translate to families, with more work needed to understand how to overcome this “novelty effect”. Families reported varied levels of engagement with the Fitbit’s partnering app. The ability to link wearable devices to a partnering app offers some benefits, as it enables users to track their long-term PA as well as their “real-time” PA [72]. It is unclear whether wearables differ in their effectiveness based on whether the user engages with its partnering app or not, and future research may benefit from directly comparing this. Despite all family members receiving a Fitbit, some reported “a collective experience” was lacking due to the inability to link multiple devices to the same device/app. Studies have found that parent–child co-participation in PA is associated with higher PA levels in adolescents [73,74]. Therefore, utilising wearable brands or models that enable interconnectivity between devices may encourage families to be active together. Child-friendly Fitbit devices, such as the Ace 2 and 3, allow this interconnectivity by linking devices with an adult device via the app’s “family account” [42]. Future research may benefit from using these devices (or similar).

### 4.3. Wearable Ease of Use 

Most family members reported the Fitbit was easy to use; however, there were varying number of family members who reported problems using the Fitbit. Difficulties charging, syncing, or setting up the devices were reported. In Drehlich et al.’s [30] study, adolescents similarly reported difficulties charging and syncing the Fitbit Flex. According to the TAM [24], a wearable’s ease of use could play a critical role in long-term wearable use [24]. Studies in adults have found that a wearable’s ease of use can predict wearable use and acceptability [27,75]. For example, a study by Rupp et al. [75] investigated what impacts adults’ (18 to 83 years) intention to continue using a wearable they were provided with by the research team. It was found that the wearable’s usability was significantly associated with user’s perception that the wearable could help them achieve their fitness goals (usefulness) and their intention to continue using the device [75]. A novel finding from the current study was families’ difficulties understanding what some PA outputs (e.g., “fire symbol” or active minutes) meant. This may limit the wearable’s usefulness for increasing awareness of PA levels. Researchers must consider a wearable’s ease of use and consider ways to support families’ wearable use, such as providing instructions on how to complete maintenance tasks (syncing, charging) and how to use and understand features.

### 4.4. Wearable Usefulness

The ActiGraph data demonstrated a slight increase in the number of family members meeting PA guidelines after using the Fitbit (but still 60–67% of the sample; Table 7). This reflects the mixed evidence found in recent systematic reviews exploring wearables’ impact on child [13] and adult [76] PA. The qualitative data reflected differences in families’ perceived changes in PA levels. When families believed the Fitbit increased PA levels, this was via three potential mechanisms of action: (1) competition and comparison, (2) monitoring and goal setting, and (3) prompts and reinforcement. Several studies have demonstrated PA competitions can increase PA, such as step count and MVPA, in adults [77,78] and children [79]; similarly, PA interventions using goal setting and self-monitoring have found promising results [80,81]. However, potential limitations of these mechanisms were acknowledged by families. Some children did not enjoy competitions, nor did all parents and children find the Fitbit’s PA prompts effective. This highlights the potential importance of tailoring future intervention components to individual families or providing them with the flexibility (like this study) to use wearables in ways that work best for them. A novel finding of the current study was the number of parents who were confident their child was meeting PA guidelines increased after using the Fitbit. The current study and other similar studies [82] have found that wearables can increase awareness of PA levels, with some studies advocating the importance of increasing parents’ awareness of their child’s PA levels [83]. However, families were unsure of PA guidelines and whether (and how) the Fitbit’s outputs (e.g., steps, heart rate, active minutes) were equivalent to guidelines. When considering future interventions, providing additional materials, such as written materials containing PA recommendations [84], may support families’ understanding (if the intention is to increase knowledge or understanding). The Fitbit was not only deemed “useful” for PA-related behaviours but also changing sleep and eating behaviours, meaning they may be incorporated into intervention targeting additional outcomes (e.g., sleep and obesity).

### 4.5. Attitudes towards Using Wearables

Most family members enjoyed using the Fitbit (particularly children). However, parents reported an increase in stress using the Fitbit, which was attributed to its usability as well as the Fitbit’s ability to accurately capture their PA. Validation studies have reported mixed evidence regarding wearables accuracy to capture PA, such a step count and MVPA [85,86]. Perceived accuracy of a wearable has been found to impact adults’ acceptance of using the device [87] and the intention to use one in the future [88]. Rupp et al. [75] explored adults’ trust in their wearable device. “Wearables trust” was sub-divided into five categories: (1) privacy, (2) validity, (3) reliability, (4) system capabilities (the device has relevant features), and (5) system transparency (easy to determine the device limitations and understand the methods it uses to calculate information) [75]. It was found that the device’s usability was associated with users’ perceived trustworthiness of the device, and this impacted intention to use the device again [75]. The Fitbit’s inability to measure and monitor certain types of activities (e.g., weightlifting), which has also been found in validation studies [89], may discourage individuals who enjoy these activities from using wearables or continuing to participate in their desired activities (e.g., replacing these with “step-based” activities). Additionally, some families reported the wearable caused skin irritation. The cause and extent of this is unclear, but other studies have also reported skin reactions to other Fitbit [90] and wearable [91] devices. Thus, setting expectations about wearables’ capabilities as well as addressing usability issues may create more positive experiences using wearables in the family.

### 4.6. Intentions to Use Wearables Again and Intervention Suggestions

Most adults agreed they would consider purchasing a Fitbit or similar device, for themselves and children and were willing to incorporate a wearable into their child’s daily routine. This demonstrates wearables acceptance in behaviour-change research. However, adults would prefer to use a more “feature-rich” device that could allow them to incorporate the wearable further within their daily lives (e.g., answering phone calls), and children may prefer using a waterproof device with a gamification element. Other studies have found parents favour waterproof wearables for their child [43], and including gamification components (e.g., avatar models) alongside wearable use may increase child PA [92]. An interesting finding was that some parents and children felt they were too young to use a wearable. The young age group in this study was of interest, given most studies have focused on adolescents (10 to 19 years) [13] and given evidence that child PA levels decline around the age of 8 years [93]. However, this finding suggests that using wearables in younger children may not be acceptable. Exploring families’ suggestions for future wearable interventions provides some insight into how wearables can be utilised in this younger age group. Intervention suggestions included competition, self-monitoring, goal setting, and tailoring intervention components, which corresponded with identified mechanisms of action (Pillar 6) and external variables (e.g., individual differences) influencing wearables impact on PA (Pillars 1 and 2). Thus, this study may provide the basis for future family-based wearable intervention components, but their acceptance and feasibility for implementation must be explored further.

### 4.7. Implications for Intervention Development

The findings from this study will be used to inform the development of a future family-based wearable intervention by considering families’ suggestions and integrating the findings into intervention development frameworks, such as the Behaviour-Change Wheel (BCW; [33]). Integrating TDF findings as part of the BCW’s intervention development process will enable a future family-based wearable intervention to be developed from this study’s findings considering the intervention’s functions and components, for example, considering how to overcome environmental barriers (e.g., work and school; Pillar 2) via environmental restructuring (one of the BCW’s intervention functions) [33]. The findings from this study can also be used to anticipate wearable acceptability [21] and consider techniques to overcome potential barriers (e.g., creating a collective experience, charging and syncing difficulties, understanding of wearable outputs).

Future work will continue to engage families in the intervention development process and consider their perspectives for overcoming identified barriers alongside opinions on proposed intervention components identified in this study. Considering the intervention’s target users (families) experience may potentially increase the effectiveness of wearables (which to date have been mixed [13]) and create more sustainable increases in PA [94].

### 4.8. Strengths and Limitations

A strength of this study was the integration of mixed-methods findings using the PIP, which identified similarities and differences in quantitative and qualitative findings [41]. This is the first study to use this approach to inform the acceptability of using wearables in families (and children [13]) and provides a greater understanding of the dynamic processes behind wearable acceptability, which may not be captured using quantitative methods alone. Additional strengths included all family members receiving the wearable (which has not been done in some studies [43]), focus on younger children (most studies have included adolescents [13]), and the diversity of included families, such as the higher levels of deprivation (based on IMD) of the study’s participants, which may highlight wearables use for reducing health inequalities [95]. Limitations of this study include the low ActiGraph compliance rates (Section 3.1); however, this highlights that future research must consider ways to improve compliance rates. Similarly, future research may benefit more from using accelerometer’s raw data (e.g., using open-source packages; [96]) than activity counts (as in this study) to determine PA levels, as this may facilitate harmonisation between studies [96,97]. Additionally, most parents currently or had previously owned a wearable, which may reflect pre-existing motivation to monitor/increase their PA levels and their willingness for their child(ren) to use the Fitbit.

## 5. Conclusions

To conclude, this study provides insights into families’ acceptability of using wearables, outlining families’ experiences, key barriers, and facilitators of using wearables and intervention suggestions. Overall, Fitbits were considered easy to use, but researchers must consider ways to improve their usefulness for increasing PA, such as overcoming external barriers to PA (e.g., in other settings such as work and school). This study highlights ways wearables may be integrated into future family-based PA interventions by utilising theoretical frameworks and integrating mixed-methods findings.

## Figures and Tables

**Table 1:** A worked example (data taken from a well-being mentor intervention study) of the Pillar Integration Process, taken from Johnson et al. [41].

Quantitative Data	Quantitative Categories	Pillar	Qualitative Categories	Qualitative Codes
Response rate (%) School 1: 85 School 2: 35 School 3: 35 School 4: 60 School 5: 100 School 6: 85 School 7: 60 School 8: 20	Heterogeneity between schools in number of returns and completeness of returns (response rate ranges from 20% to 100%)	Compatibility of setting, staff, and intervention	Compatibility of context (school) and program required flexibility to account for school autonomy	‘‘Schools are very autonomous and that’s often very difficult for partners who aren’t in education to understand. You can’t tell them what to do. So, there was variation.’’

**Table 2:** Participant demographics.

	Adults (*n =* 36) ^a^	Target Children (*n =* 29)	Siblings (*n =* 12) ^b^
Age			
Mean (SD)	38 (7.7) ^a^	6 (1.5)	10 (3.9)
Range	24–55 years	5–9 years	3–17 years
Sex, *n* (%)			
Male	12 (33.3%)	15 (51.7%)	6 (50%)
Female	24 (66.7%)	14 (48.3%)	6 (50%)
Ethnicity, *n* (%)			
White British	24 (66.7%)	17 (58.6%)	5 (41.7%)
Pakistani Heritage	8 (22.2%)	9 (31%)	5 (41.7%)
Mixed: White and Black Caribbean	2 (5.6%)	2 (6.9%)	2 (16.7%)
Mixed: White and Chinese	1 (2.8%)	0	0
Mixed: Pakistani and Indian	1 (2.8%)	0	0
Mixed: White and Asian (Pakistani and Indian)	0	1 (3.4%)	0
Wearable ownership, *n* (%)			
Currently own	11 (30.6%)	4 (13.8%)	2 (16.7%)
Duration of use, *n* (%)			
<1 month	0	1 (25%)	0
1–5 months	1 (9%)	0	1 (50%)
6–11 months	0	1 (25%)	1 (50%)
1–2 years	3 (27%)	1 (25%)	0
>2 years	7 (64%)	1 (25%)	0
Previously owned	9 (25%)	0	1 (8.3%)
Never owned	16 (44.4%)	25 (86.2%)	9 (75%)
Index of Multiple Deprivation (IMD) decile ^c^, *n* (%)			
Decile 1–3	11 (45.8%)		
Decile 4–7	9 (37.5%)		
Decile 8–10	4 (16.7%)		
Employment status, *n* (%)			
Full-time employed	20 (55.6%)		
Part-time employed	7 (19.4%)		
Self-employed	6 (16.7%)		
Unemployed/Stay-at-home parent	2 (5.6%)		
Long-term sick leave	1 (2.8%)		
Highest educational qualification ^d^, *n* (%)			
General Certificate of Secondary Education (GCSE)	4 (11.1%)		
Advanced level (A level)	8 (22.2%)		
National Vocational Qualification (NVQ) level 4	7 (19.4%)		
Bachelor’s degree	13 (36.1%)		
Master’s degree	4 (11.1%)		

^a^ Includes parents, stepparents, and grandmother, except adult mean (SD) age and age range, where the grandmother was not included. Grandmother was 53 years. ^b^ Includes siblings and cousins. ^c^ IMD based on home postcode (relevant for target children and siblings). ^d^ Qualification listed or equivalent (qualifications refer to those taken in England).

**Table 3:** Themes and their corresponding Theoretical Domains Framework (TDF) component, developed using the thematic analysis to reflect the use and acceptance of wearables.

Theoretical Domains Framework (TDF) Component Definition	Thematic Themes ^a^
**Identity (as part of the “social/professional role and identity” component)** Personal qualities of an individual	1. The Fitbit’s impact on physical activity is influenced by family member’s pre-Fitbit physical activity levels
**Environmental context and resources** Any circumstance of a person’s situation or environment that discourages or encourages the development of skills and abilities, independence, social competence, and adaptive behaviour	2. School and work as barriers of Fitbit use and physical activity 3. COVID-19 restrictions as a barrier of physical activity
**Behavioural regulation** Anything aimed at managing or changing observed or measured actions	4. The extent of Fitbit use 5. Monitoring and goal setting as mechanisms of action 6. Suggestions for future interventions: tailor towards the individual and their routine
**Social influences** Interpersonal processes that can cause an individual to change their behaviour	7. Individual and collective Fitbit use 8. Competition and comparison as mechansisms of action 9. Suggestions for future interventions: competition
**Emotion** A complex reaction pattern, involving experiential, behavioural, and physioloical elements	10. Fitbit usability 11. Fitbit aesthetics impacts enjoyment of using the Fitbit
**Knowledge** An awareness of the existence of something	12. Interpretation of Fitbit outputs 13. The Fitbit’s (in)ability to improve understanding of physical activity guidelines 14. The Fitbit increased awareness of physical activity levels 15. Suggestions for future interventions: incorporate more physical acitivty information
**Beliefs about consequences** Acceptance of the truth, reality, or valdity about outcomes of a behaviour in a situation	16. The influence of the Fitbit on physical activity 17. The Fitbit’s impact on health outcomes other than physical actvity (e.g., sleep and diet) 18. The Fitbit’s abiltiy to capture physical activity
**Reinforcement** Increasing the probability of a response by arranging a dependent relationship or contingency between the response and stimulus	19. Prompts and reinforcement as mechanisms of action
**Decision processes (as part of the “memory, attention, and decision processes” component)** Choose between two or more alternatives	20. Intentions and considerations for future wearable use 21. Use of the Fitbit’s features and partnering application

^a^ Numbers represent the theme number, which are referred to throughout the Pillar Integration Process results (Section 3.4).

## Data Availability

The data presented in this study are available on request from the corresponding author. The data are not publicly available due to anonymity and confidentiality considerations.

## Supplementary Materials

The following supporting information can be downloaded at: https://www.mdpi.com/article/10.3390/ijerph19063472/s1. Table S1: The Theoretical Domains Framework (TDF) questionnaire items; Table S2: Weekly survey items, Table S3: The number (%) of adult participants rating their families experiences of using the Fitbit as ‘Poor’, ‘Fair’, ‘OK’, ‘Good’ and ‘Excellent’ (results from the weekly surveys); Table S4: The number (%) of family members agreeing (or strongly agreeing) to statements on the weekly surveys regarding Fitbit acceptance; Table S5: Parent and child’s perceived capability, opportunity, and motivation to use a wearable and participate in PA (results from the TDF questionnaire.
