# Supplementary material for: Exploring Families’ Acceptance of Wearable Activity Trackers: A Mixed-Methods Study
Source: Int J Environ Res Public Health. 2022 Mar 15;19(6):3472. doi: 10.3390/ijerph19063472 (PMC8950917; doi:10.3390/ijerph19063472)
Supplement: Supplementary file 1 [file ijerph-19-03472-s001.zip › Creaser_Supplementary material 2.pdf]

A family-based mixed methods acceptability study of wearable activity trackers, in 5- to 9-year-old children

Supplementary materials

Supplementary Table S2. Weekly survey items.

**Perceived ease of use**
I/my child found the Fitbit is easy to use
I/my child found the Fitbit uncomfortable to wear
I have/my child has experienced problems with the Fitbit
**Perceived usefulness**
The Fitbit motivates me/my child to be more active
I am/my child is more active because of the Fitbit
As a family, we are more active because of the Fitbit[a]
**Attitudes towards use**
How would you rate your families experience using the Fitbit?[a]
I like/my child likes wearing the Fitbit
I am/my child is embarrassed to wear the Fitbit
**Intention to use**
I would consider purchasing a Fitbit, or similar device, for myself/my child after this study
