# Supplementary material for: Exploring Families’ Acceptance of Wearable Activity Trackers: A Mixed-Methods Study
Source: Int J Environ Res Public Health. 2022 Mar 15;19(6):3472. doi: 10.3390/ijerph19063472 (PMC8950917; doi:10.3390/ijerph19063472)
Supplement: Supplementary file 1 [file ijerph-19-03472-s001.zip › Creaser_Supplementary material 3.pdf]

A family-based mixed methods acceptability study of wearable activity trackers, in 5- to 9-year-old children

Supplementary materials

Supplementary Table S3. The number (%) of adult participants rating their families experiences of using the Fitbit as 'Poor', 'Fair', 'OK', 'Good' and 'Excellent' (results from the weekly surveys)

	Poor	Fair	OK	Good	Excellent
**Week 1** (*n*=34)	0	3 (9%)	9 (26%)	14 (41%)	8 (24%)
**Week 2** (*n*=34)	0	0	10 (29%)	16 (47%)	8 (24%)
**Week 3** (*n*=33)	0	0	9 (27%)	15 (45%)	9 (27%)
**Week 4** (*n*=33)	0	1 (3%)	6 (18%)	13 (39%)	13 (39%)
