# Supplementary material for: Exploring Families’ Acceptance of Wearable Activity Trackers: A Mixed-Methods Study
Source: Int J Environ Res Public Health. 2022 Mar 15;19(6):3472. doi: 10.3390/ijerph19063472 (PMC8950917; doi:10.3390/ijerph19063472)
Supplement: Supplementary file 1 [file ijerph-19-03472-s001.zip › Creaser_Supplementary material 4.pdf]

A family-based mixed methods acceptability study of wearable activity trackers, in 5- to 9-year-old children

Supplementary materials.

Supplementary Table S4. The number (%) of family members agreeing (or strongly agreeing) to statements on the weekly surveys regarding Fitbit acceptance. n/N (%)

	Week 1			Week 2			Week 3			Week 4		
**Question**	**Adults**	**Target children**	**Siblings**	**Adults**	**Target children**	**Siblings**	**Adults**	**Target children**	**Siblings**	**Adults**	**Target children**	**Siblings**
**Perceived ease of use**												
**Easy to use**	28/34 (82%)	17/28 (61%)	9/12 (75%)	29/34 (85%)	17/27 (63%)	7/11 (64%)	27/34 (79%)	21/27 (78%)	10/11 (91%)	27/33 (82%)	18/27 (67%)	8/11 (73%)
**Uncomfortable to wear**	10/34 (29%)	6/28 (21%)	3/12 (25%)	7/34 (21%)	3/27 (11%)	6/11 (55%)	9/34 (26%)	8/27 (30%)	7/11 (64%)	6/33 (18%)	7/27 (26%)	4/11 (36%)
**Experienced problems**	9/34 (26%)	4/28 (14%)	5/12 (42%)	7/34 (21%)	3/27 (11%)	5/11 (45%)	7/34 (21%)	2/27 (7%)	3/11 (27%)	10/33 (30%)	7/27 (26%)	4/11 (36%)
**Perceived usefulness**												
**More motivated to be active**	20/34 (59%)	19/28 (68%)	9/12 (75%)	18/34 (53%)	23/27 (85%)	9/11 (82%)	21/34 (62%)	23/27 (85%)	8/11 (73%)	19/33 (58%)	24/27 (89%)	9/11 (82%)
**More active**	17/34 (50%)	17/28 (61%)	9/12 (75%)	17/34 (50%)	21/27 (78%)	8/11 (73%)	18/34 (53%)	21/27 (78%)	7/11 (64%)	17/33 (52%)	22/27 (81%)	8/11 (73%)
**More active as a family**	21/34 (62%)	n/a	n/a	21/34 (62%)	n/a	n/a	22/34 (65%)	n/a	n/a	24/33 (73%)	n/a	n/a
**Attitudes towards use**												

**Likes wearing**	25/34 (74%)	24/28 (86%)	8/12 (67%)	24/34 (71%)	26/27 (96%)	7/11 (64%)	25/34 (74%)	24/27 (89%)	6/11 (55%)	24/33 (73%)	24/27 (89%)	8/11 (73%)
**Embarrassed to wear**	0/34 (0%)	0/28 (0%)	0/12 (0%)	0/34 (0%)	0/27 (0%)	0/11 (0%)	0/34 (0%)	0/27 (0%)	0/11 (0%)	0/33 (0%)	1/27 (4%)	0/11 (0%)
**Intention to use**												
**Consider purchasing Fitbit or similar device**	21/33 (64%)	20/28 (71%)	8/12 (67%)	22/32 (69%)	20/28 (71%)	6/12 (50%)	25/33 (76%)	21/28 (78%)	6/12 (50%)	23/33 (70%)	20/28 (71%)	5/12 (42%)
