# Supplementary material for: Exploring Families’ Acceptance of Wearable Activity Trackers: A Mixed-Methods Study
Source: Int J Environ Res Public Health. 2022 Mar 15;19(6):3472. doi: 10.3390/ijerph19063472 (PMC8950917; doi:10.3390/ijerph19063472)
Supplement: Supplementary file 1 [file ijerph-19-03472-s001.zip › Creaser_Supplementary matieral 5.pdf]

A family-based mixed methods acceptability study of wearable activity trackers, in 5- to 9-year-old children

Supplementary materials

Supplementary Table S5. Parent and child's perceived capability, opportunity, and motivation to use a wearable and participate in PA (results from the TDF questionnaire), n/N (%)

	Pre-Fitbit	Post-Fitbit
**Psychological Capability**		
**Parents perceived understanding of MVPA**		
No understanding	2/24 (8%)	0/22 (0%)
Little understanding	3/24 (13%)	2/22 (9%)
Some understanding	5/24 (21%)	9/22 (41%)
A lot of understanding	13/24 (54%)	8/22 (36%)
Unsure	1/24 (4%)	3/22 (14%)
**Parents understanding of PA guidelines (minutes in MVPA)**		
<60 mins	9/24 (38%)	5/22 (23%)
60 mins	10/24 (42%)	11/22 (50%)
>60 mins	1/24 (4%)	2/22 (9%)
Unsure	4/24 (17%)	4/22 (18%)
**Child is achieving PA guidelines**		
Not at all confident	1/29 (3%)	0/26 (0%)
Not confident	2/29 (7%)	0/26 (0%)
Confident	16/29 (55%)	20/26 (77%)
Very confident	3/29 (10%)	4/26 (15%)
Unsure	7/29 (24%)	2/26 (8%)
**Physical Capability**		
**Child has the physical abilities to be active**		

Not at all confident	0/29 (0%)	0/26 (0%)
Not confident	0/29 (0%)	0/26 (0%)
Confident	10/29 (34%)	9/26 (35%)
Very confident	19/29 (66%)	17/26 (65%)
Unsure	0/29 (0%)	0/26 (0%)
**Parent has the physical abilities to support their child being active**		
Not at all confident	0/24 (0%)	0/22 (0%)
Not confident	1/24 (4%)	1/22 (5%)
Confident	13/24 (54%)	11/22 (50%)
Very confident	10/24 (42%)	9/22 (41%)
Unsure	0/24 (0%)	1/22 (5%)
**Social Opportunity**		
**Child has someone to be active with**		
Not at all confident	0/29 (0%)	0/26 (0%)
Not confident	1/29 (3%)	1/26 (4%)
Confident	19/29 (66%)	15/26 (58%)
Very confident	9/29 (31%)	7/26 (27%)
Unsure	0/29	0/26
**Physical Opportunity**		
**Child has the facilities to be active**		
Not at all confident	0/29 (0%)	2/26 (8%)
Not confident	4/29 (14%)	1/26 (4%)
Confident	15/29 (52%)	17/26 (65%)
Very confident	8/29 (28%)	5/26 (19%)
Unsure	2/29 (7%)	1/26 (4%)
**Child has enough space to be active**		
Not at all confident	0/29 (0%)	2/26 (8%)
Not confident	2/29 (7%)	1/26 (4%)
Confident	15/29 (52%)	15/26 (58%)
Very confident	12/29 (41%)	8/26 (31%)
Unsure	0/29 (0%)	0/26 (0%)

**Child has enough time to be active**		
Not at all confident	0/29 (0%)	0/26 (0%)
Not confident	1/29 (3%)	1/26 (4%)
Confident	11/29 (38%)	14/26 (54%)
Very confident	11/29 (38%)	6/26 (23%)
Unsure	6/29 (21%)	5/26 (19%)
**Automatic Motivation**		
**Change in parent/guardian stress**		
A large decrease	0/24 (0%)	0/22 (0%)
A small decrease	2/24 (8%)	1/22 (5%)
No change	15/24 (63%)	13/22 (59%)
A small increase	5/24 (21%)	6/22 (27%)
A large increase	2/24 (8%)	2/22 (9%)
**Change in child's stress**		
A large decrease	0/29 (0%)	1/26 (4%)
A small decrease	3/29 (10%)	2/26 (8%)
No change	19/29 (66%)	18/26 (69%)
A small increase	5/29 (17%)	5/26 (19%)
A large increase	2/29 (7%)	0/26 (0%)
**Reflective Motivation**		
**PA's perceived impact on physical health**		
No impact	0/24 (0%)	0/22 (0%)
Little impact	0/24 (0%)	0/22 (0%)
Some impact	2/24 (8%)	1/22 (5%)
A large impact	22/24 (92%)	21/22 (95%)
Unsure	0/24 (0%)	0/22 (0%)
**PA's perceived impact on mental health**		
No impact	0/24 (0%)	0/22 (0%)
Little impact	1/24 (4%)	0/22 (0%)
Some impact	2/24 (8%)	1/22 (5%)
A large impact	20/24 (83%)	21/22 (95%)

Unsure	1/24 (4%)	0/22 (0%)
**PA's perceived impact on academic attainment**		
No impact	0/24 (0%)	0/22 (0%)
Little impact	1/24 (4%)	0/22 (0%)
Some impact	9/24 (38%)	10/22 (45%)
A large impact	10/24 (42%)	11/22 (50%)
Unsure	4/24 (17%)	1/22 (5%)
**PA's perceived impact on social development**		
No impact	0/24 (0%)	0/22 (0%)
Little impact	1/24 (4%)	0/22 (0%)
Some impact	7/24 (29%)	6/22 (27%)
A large impact	15/24 (63%)	16/22 (73%)
Unsure	1/24 (4%)	0/22 (0%)
**Wearable can increase child's PA**		
Not at all confident	2/29 (7%)	0/26 (0%)
Not confident	1/29 (3%)	1/26 (4%)
Confident	12/29 (41%)	16/26 (62%)
Very confident	2/29 (7%)	5/26 (19%)
Unsure	12/29 (41%)	4/26 (15%)
**Beneficial to learn more about child's PA levels**		
Not at all beneficial	0/29 (0%)	0/26 (0%)
Not beneficial	1/29 (3%)	0/26 (0%)
Beneficial	17/29 (59%)	16/26 (62%)
Very beneficial	10/29 (34%)	10/26 (38%)
Unsure	1/29 (3%)	0/26 (0%)
**Willingness to incorporate more PA into child's routine**		
Not at all willing	0/29 (0%)	0/26 (0%)
Unwilling	0/29 (0%)	0/26 (0%)
Willing	20/29 (69%)	11/26 (42%)
Very willing	8/29 (28%)	13/26 (50%)
Unsure	1/29 (3%)	2/26 (8%)
**Willingness to incorporate a wearable into child's routine**		

Not at all willing	0/29 (0%)	0/26 (0%)
Unwilling	1/29 (3%)	0/26 (0%)
Willing	11/29 (38%)	10/26 (38%)
Very willing	13/29 (45%)	13/26 (50%)
Unsure	4/29 (14%)	3/26 (12%)
