# Supplementary material for: Exploring Families’ Acceptance of Wearable Activity Trackers: A Mixed-Methods Study
Source: Int J Environ Res Public Health. 2022 Mar 15;19(6):3472. doi: 10.3390/ijerph19063472 (PMC8950917; doi:10.3390/ijerph19063472)
Supplement: Supplementary file 1 [file ijerph-19-03472-s001.zip › Creaser_Supplemetary material 1.pdf]

**A family-based mixed methods acceptability study of wearable activity trackers, in 5- to 9-year-old children**

## Supplementary materials

## Supplementary Table S1. The Theoretical Domains Framework (TDF) questionnaire items

| Component of the COM-B model | Component of the TDF | Question(s) |
|---|---|---|
| Psychological Capability | Knowledge (Perceived) | • How much do you understand the term "moderate-to-vigorous physical activity"?<br>• How confident are you that your child is achieving physical activity guidelines? |
| | Knowledge (Actual) | • How many minutes of moderate-to-vigorous physical activity should children participate in per day? |
| Physical Capability | Physical skills | • How confident are you that, typically:<br>your child has the physical ability to be active?<br>you have the physical ability to promote your child's physical activity? |
| Social Opportunity | Social influences | • How confident are you that, typically, your child has someone to be physically active with? |
| Physical Opportunity | Environmental context and resources | • How confident are you that your child:<br>has the facilities to be physically active?<br>has enough space to be physically active?<br>has enough time to be physically active? |
| Automatic motivation | Emotion | • How much do you think incorporating a wearable device (e.g. Fitbit) into your child's day would:<br>affect your stress?<br>affect your child's stress? |
| Reflective motivation | Optimism | • How confident are you that wearable activity trackers can increase your child's physical activity? |
| | Beliefs about consequences | • How beneficial would it be for you to learn more about your child's physical activity levels?<br>• To what extent do you think physical activity impacts a child's:<br>Physical health<br>Mental health<br>Academic attainment<br>Social development |
| | Intentions | • How willing would you be to:<br>incorporate more physical activity into your child's daily routine?<br>incorporate wearable activity trackers into your child's daily routine? |
